# Does Exsanguination Enhance Skin Microcirculation in Remote Ischemic Conditioning Using a Tourniquet? A Randomized Controlled Trial on 50 Healthy Subjects

**DOI:** 10.1055/a-2779-2935

**Published:** 2026-03-27

**Authors:** Carola Rist, Jens Rothenberger, Adrien Daigeler, Andrea Wenger

**Affiliations:** 1 Department of Hand, Plastic, Reconstructive and Burn Surgery, BG Clinic Tuebingen, Eberhard Karls University Tuebingen, Tuebingen, Germany

**Keywords:** remote ischemic conditioning, ischemia, reperfusion, exsanguination, tourniquet

## Abstract

**Background:**

Remote Ischemic Conditioning (RIC) improves cutaneous microcirculation in free flap surgery. As no universal RIC protocol exists, refinements have mainly focused on cycle duration and number. We aimed to assess whether ischemic stimulus intensity, using a tourniquet with additional exsanguination, affects cutaneous microcirculation.

**Methods:**

In this randomized controlled trial, 50 healthy volunteers were randomized into two groups (25 each). Both underwent an RIC protocol of three cycles of 10-minute ischemia followed by 10-minute reperfusion. In the control group (Tourniquet, T), ischemia was induced with a surgical tourniquet inflated to 250 mm Hg on the right upper arm. In the experimental group (Tourniquet with exsanguination, T ^e^ ), the arm was additionally exsanguinated using an Esmarch bandage. Cutaneous microcirculation parameters (oxygen saturation [SO _2_ ], blood flow [BF], and relative amount of hemoglobin [rHb]) were assessed non-invasively with the oxygen to see (O2C) device.

**Results:**

Both groups showed significant changes in all microcirculatory parameters compared with baseline. Although SO _2_ and BF values tended to be higher during reperfusion in the experimental group (T ^e^ ), overall differences were not statistically significant (except at the end of the first reperfusion phase in BF [T _mean_ 1.33 ± 0.54 vs. T ^e^_mean_ 1.93 ± 0.91; *p* = 0.017] and at the beginning of the second reperfusion phase in SO _2_ [T _mean_ 1.05 ± 0.18 vs. T ^e^_mean_ 1.13 ± 0.17; *p* = 0.045], respectively).

**Conclusion:**

Exsanguination, in addition to tourniquet application, does not enhance cutaneous microcirculation in RIC protocols. The relatively small sample size remains a limitation and restricts generalizability. Future studies should confirm these findings in larger and more diverse cohorts and explore potential clinical applications.

## Introduction

Significant therapeutic challenges for physicians arise from ischemia–reperfusion injury after trauma (e.g., finger replantation) and reconstructive surgery. 1 Ischemia–reperfusion injury is a complex medical condition in which the abrupt rise of oxygen levels causes excessive production of reactive oxygen species 2 3 4 and increases the release of proinflammatory mediators. 2 5 Thereby, reperfusion itself can cause endothelial dysfunction and secondary injury 6 with cell damage leading to necrosis and subsequent flap loss.

One strategy to induce systemic protection against ischemia–reperfusion injury is Remote Ischemic Conditioning (RIC). RIC is the application of transient, repeated, non-harmful cycles of ischemia and reperfusion to a vascular territory remote to the target tissue. The underlying mechanisms of RIC are not yet fully understood, but it generates a systemic release of humoral factors 7 8 9 and activates nerve fibers, resulting in a secondary release of messengers remote to the initial application site of the ischemic stimulus. 10 11 12 Also, suppressing the immune-inflammatory response is an essential mechanism of RIC. 13

Since its discovery by Przyklenk et al in 1993, 14 RIC has been the subject of research in various disciplines, most pronounced in cardiology. In the field of plastic and reconstructive surgery, it was initially shown in animal studies that microcirculation in adipocutaneous as well as muscle flaps could be improved by RIC. 15 Kharbanda et al, in 2002, were the first to show in 14 healthy volunteers that RIC, by inflating a tourniquet on the forearm, can protect the contralateral arm from endothelial dysfunction after ischemia and reperfusion. 16 RIC can be applied before, during, and after persistent ischemia (i.e., remote ischemic pre-, peri-, and postconditioning), although remote ischemic postconditioning was slightly less effective in comparison. 17

Previously published articles yielded promising results in improving cutaneous microcirculation in humans. 18 19 20 However, they are heterogeneous in terms of the applied RIC protocol, especially with regard to the number and duration of the ischemia cycles. Usually, three to four ischemia and reperfusion intervals of 5 to 10 minutes each have been described. 21 Due to the findings of Kolbenschlag et al 20 and Sogorski et al, 22 a protocol consisting of three ischemia and reperfusion intervals of 10 minutes each is commonly used. Other types of stimulus application, such as nerve stimulation by electrical stimulation and applying capsaicin, could also show an effect comparable to ischemic conditioning in animal models. 23 24 Recent studies have explored various modifications to RIC protocols, including different ischemia durations and stimulation methods, to optimize their clinical application. In 2015, Kolbenschlag et al proved in a randomized trial of 40 healthy volunteers that RIC of the upper extremity is superior to that of the lower extremity, 19 and therefore the ischemic tissue mass has little impact on cutaneous blood flow (BF). To our knowledge, no previous study has investigated the intensity of the ischemic stimulus of the upper extremity. We, therefore, conducted the following study to test the hypothesis that additional exsanguination can further enhance cutaneous microcirculation in RIC compared with the tourniquet alone.

## Methods

The study was approved by the local ethics committee of the Eberhard Karls University of Tuebingen (registration number: 419/2017BO2) and performed in accordance with the Declaration of Helsinki.

**Fig. 1:**
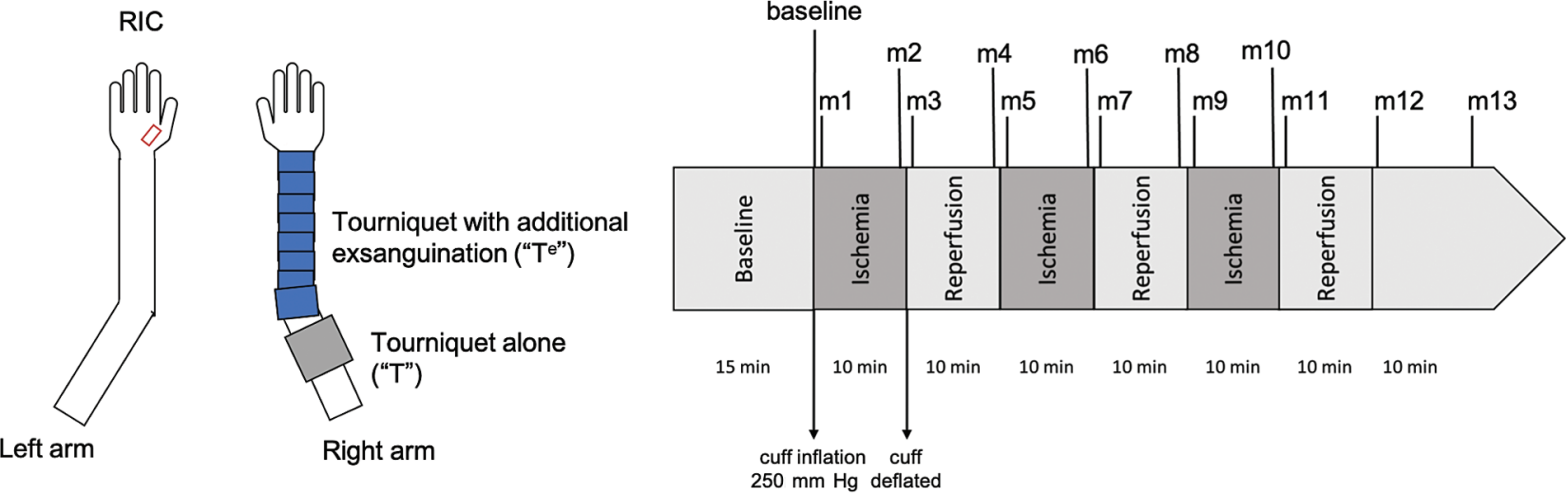
Study design: Application of the tourniquet on the right arm with or without exsanguination (T ^e^ , experimental group; T, control group), taking measurements with the O2C device on the left hand (RIC, remote ischemic conditioning). Baseline measurements after 15 minutes of acclimatization (baseline), inflation (250 mm Hg), and deflation of the cuff in three cycles, 10 minutes each, with repeated measurements at the beginning and end of each ischemia and reperfusion phase (m1–m12) as well as 10 minutes after the last reperfusion (m13). O2C, oxygen to see device.

For this randomized controlled trial, we recruited 50 young, healthy volunteers without a history of chronic illnesses or current medication. In advance, we instructed the subjects to refrain from caffeine, cocoa, and other foods that affect blood circulation and nicotine at least 12 hours before the measurements. General characteristics were documented ( Table 1 ), and the subjects were randomized into two groups: “Tourniquet” ([T], control group, *n* = 25) and “Tourniquet with exsanguination” ([T ^e^ ], experimental group, *n* = 25). In each group, the conditioning protocol consisted of three cycles of 10 minutes ischemia followed by 10 minutes of reperfusion as recommended by Kolbenschlag et al. 23 25 Ischemia in the control group T was induced by a surgical tourniquet (Ulrich Medical, Ulm, Germany) inflated to 250 mm Hg on the right upper arm. Additionally, in the experimental group T ^e^ , the arm was exsanguinated with an Esmarch bandage before inflating the tourniquet ( Fig. 1 ).

**Table 1:** Patient's general characteristics according to each study group

	Control (T)	Experimental group (T ^e^ )
Age (years), mean ± SD	26.24 ± 3.65	25.64 ± 2.71
Gender, M/F	13/12	13/12
Height (m), mean ± SD	175.08 ± 10.28	175.08 ± 8.07
Weight (kg), mean ± SD	67.20 ± 8.01	71.32 ± 12.85
BMI (kg/m ^2^ ), mean ± SD	21.92 ± 1.93	23.12 ± 2.79
Smoker, *n* (%)	2 (8%)	3 (12%)
Heart rate (bpm), mean ± SD	63.21 ± 10.41	61.53 ± 9.56
SpO _2_ (%), mean ± SD	98.92 ± 1.36	98.47 ± 1.47

Abbreviations: BMI, body mass index; M/F, male/female; SD, standard deviation; SpO _2_ , peripheral oxygen saturation during measurements.

All actions and measurements were performed by the same investigator (C.R.). Block randomization was performed with Microsoft Excel 2019, version 16.28. Blinding for either the investigator or the subjects was not possible due to the study design. For measurements of cutaneous microcirculation, we used combined white light spectroscopy (500–600 nm) and laser Doppler method (820 nm) by the O2C device (©oxygen to see, LEA Medizintechnik, Giessen, Germany, CE-certified, type LW1212, serial number 182-327-11) on the left hand in the space between the first and second metacarpal bone.

After conducting a white balance, we measured the relative amount of hemoglobin (rHb), the oxygen saturation of hemoglobin (SO _2_ ), and the relative BF. The O2C device analyzes measurements at a depth of 2 mm, reflecting the cutaneous microcirculation. The rHb reflects the total hemoglobin content in the observed tissue volume. It provides information on both microvascular density and the degree of vascular filling. The measured SO _2_ indicates the oxygenation status of hemoglobin at the venous end of the capillary network. Relative BF is determined by multiplying the velocity of BF by the number of erythrocytes traveling at each velocity, followed by summation across all detected velocity ranges. This represents the perfusion rate on the microvascular network, serving as an indicator of blood supply within the analyzed tissue segment. 25 26

The subjects were placed in a supine position in a temperature-controlled room at 22 °C without direct light exposure. Baseline measurements were taken after 15 minutes of rest. Following this, the RIC protocol was applied, and the measurements were performed directly at the beginning and the end of each ischemia and reperfusion phase (m1–m10), every 10 minutes for 60 minutes (data up to 30 minutes are shown in Tables 2 3 4 ) after the last ischemia cycle (m11–m13). At each time point, we first measured the right hand (data not shown) and subsequently, the left hand (remote site). In addition to the microcirculatory values, we detected the heart rate and the peripheral oxygen saturation (SpO _2_ ) at each time point using a pulse oximetry device attached to the right great toe, so as not to interfere with the measurements on the upper extremity.

**Table 2:** Measurements of relative blood flow in the left (remote) arm

	Control (T)			Experimental group (T ^e^ )			
	Mean	SD	*p* -Value versus BL	Mean	SD	*p* -Value versus BL	*p* -Value T versus T ^e^
Baseline	1.00	–	–	1.00	–	–	–
m1	1.70	0.88	**0.001**	2.59	2.08	**0.001**	0.118
m2	1.48	1.07	**0.035**	2.20	1.46	**<0.001**	**0.010**
m3	1.18	0.61	0.145	1.91	1.41	**0.003**	0.081
m4	1.33	0.54	**0.006**	1.93	0.91	**<0.001**	**0.017**
m5	1.25	0.57	**0.037**	2.08	1.03	**<0.001**	**0.002**
m6	1.38	0.66	**0.008**	2.07	1.37	**0.001**	0.079
m7	1.18	0.46	0.068	1.87	1.50	**0.008**	0.299
m8	1.28	0.50	**0.010**	1.97	1.44	**0.003**	0.121
m9	1.34	0.72	**0.026**	2.14	1.63	**0.002**	0.105
m10	1.40	0.71	**0.010**	2.02	1.53	**0.003**	0.204
m11	1.31	0.67	**0.030**	1.71	1.43	**0.020**	0.594
m12	1.34	0.77	**0.037**	1.67	1.07	**0.005**	0.399
m13	1.34	0.64	**0.014**	1.68	1.26	**0.013**	0.734

Abbreviations: BL, baseline; SD, standard deviation.

Mean corresponds to relative changes against baseline measurements. Lines highlighted in gray reflect ischemia cycles of 10 minutes, numbers highlighted in bold reflect statistically significant differences.

**Table 3:** Measurements of oxygen saturation of hemoglobin in the left (remote) arm

	Control (T)			Experimental group (T ^e^ )			
	Mean	SD	*p* -Value versus BL	Mean	SD	*p* -Value versus BL	*p* -Value T versus T ^e^
Baseline	1	–	–	1	–	–	–
m1	1.04	0.19	0.253	1.15	0.19	**0.001**	**0.037**
m2	1.09	0.08	**<0.001**	1.17	0.16	**<0.001**	0.181
m3	1.01	0.21	0.768	1.05	0.21	0.242	0.218
m4	1.16	0.20	**<0.001**	1.20	0.18	**<0.001**	0.118
m5	1.11	0.19	**0.009**	1.22	0.19	**<0.001**	**0.037**
m6	1.16	0.14	**<0.001**	1.20	0.16	**<0.001**	0.226
m7	1.05	0.18	0.187	1.13	0.17	**0.001**	**0.045**
m8	1.16	0.15	**<0.001**	1.19	0.15	**<0.001**	0.444
m9	1.08	0.14	**0.007**	1.19	0.16	**<0.001**	**0.030**
m10	1.21	0.19	**<0.001**	1.20	0.15	**<0.001**	0.762
m11	1.11	0.20	**0.014**	1.12	0.15	**0.001**	0.528
m12	1.17	0.19	**<0.001**	1.18	0.16	**<0.001**	0.725
m13	1.16	0.17	**<0.001**	1.17	0.17	**<0.001**	0.769

Abbreviations: BL, baseline; SD, standard deviation.

Mean corresponds to relative changes against baseline measurements. Lines highlighted in gray reflect ischemia cycles of 10 minutes, numbers highlighted in bold reflect statistically significant differences.

**Table 4:** Measurements of the relative amount of hemoglobin

	Control (T)			Experimental group (T ^e^ )			
	Mean	SD	*p* -Value versus BL	Mean	SD	*p* -Value versus BL	*p* -Value T versus T ^e^
Baseline	1	–	–	1	–	–	–
m1	1.00	0.09	0.905	1.05	0.10	**0.032**	0.063
m2	1.04	0.05	**0.001**	1.07	0.09	**<0.001**	0.190
m3	0.99	0.07	0.437	1.02	0.10	0.299	0.240
m4	1.06	0.06	**<0.001**	1.10	0.09	**<0.001**	0.211
m5	1.04	0.06	**0.006**	1.11	0.10	**<0.001**	**0.009**
m6	1.07	0.06	**<0.001**	1.12	0.09	**<0.001**	**0.038**
m7	1.02	0.07	0.165	1.06	0.10	**0.015**	0.151
m8	1.06	0.06	**<0.001**	1.11	0.11	**<0.001**	0.070
m9	1.02	0.05	**0.100**	1.11	0.13	**<0.001**	**0.007**
m10	1.08	0.09	**<0.001**	1.11	0.11	**<0.001**	0.122
m11	1.03	0.09	0.095	1.05	0.12	0.059	0.438
m12	1.07	0.11	**0.003**	1.09	0.12	**0.001**	0.218
m13	1.05	0.07	**0.001**	1.08	0.10	**0.001**	0.100

Abbreviations: BL, baseline; SD, standard deviation.

Mean corresponds to relative changes against baseline measurements. Lines highlighted in gray reflect ischemia cycles of 10 minutes, numbers highlighted in bold reflect statistically significant differences.

### Statistical Analysis

All calculations were performed with SPSS for Mac (IBM Corp., Released 2016, version 24.0). Statistical testing was two-sided, and *p* < 0.05 was considered significant. The graphical representation was done with Excel for Mac (Microsoft Excel 2019, version 16.28).

For technical reasons, the absolute quantification of the cutaneous microcirculation is only possible to a limited extent due to the data output of the measured values BF and rHb in “arbitrary units.” We therefore determined relative changes in relation to the baseline measurements (1 = 100%) for each subject.

All results are given in mean ± standard deviation (SD). For descriptive statistics, we checked for normal distribution using the Shapiro–Wilk test. The patient's general characteristics were compared using a bilateral unpaired *t* -test or the non-parametric Mann–Whitney U test (MWU) when appropriate. Statistical significance within the groups was assessed by an analysis of variance (ANOVA) with repeated measurements followed by an LSD post hoc test. For intergroup comparison, we used the MWU test.

## Results

There were no significant differences between groups in terms of subjects' age (T: 26.24 ± 3.65; T ^e^ : 25.64 ± 2.71; *p* = 0.513), gender (T: 13 males, 12 females; T ^e^ : 13 males, 12 females; *p* > 0.999), body mass index (BMI; T: 21.92 ± 1.93; T ^e^ : 23.12 ± 2.79; *p* = 0.085), and the number of smokers (T: 8% vs. T ^e^ : 12%; *p* = 0.646; see Table 1 ). Heart rate and SpO _2_ appeared stable in the course of the measurements over time (data not shown). No subject had to be excluded.

### Blood Flow

BF of the remote arm significantly increased in both groups compared with baseline measurements at every time point except at the beginning of the first and second reperfusion cycle in the control group (T _m3_ : 1.18 ± 0.61; *p* = 0.145; T _m7_ : 1.18 ± 0.46; *p* = 0.068). A repeated measures ANOVA with a Greenhouse–Geisser correction determined that mean performance levels showed a statistically significant difference between BF measurements versus baseline for control group T (F [8.13, 79.44] = 2.46; *p* < 0.025), and experimental group T ^e^ (F [41.28, 233.08] = 4.25; *p* = 0.006).

**Fig. 2:**
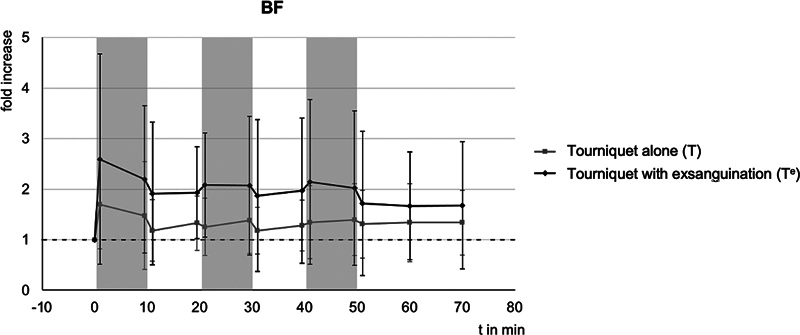
Relative changes of relative blood flow (BF) compared with baseline, with standard deviationGroup T, tourniquet, control group; Group T ^e^ , tourniquet with exsanguination, experimental group; - - -, baseline (1 = 100%), gray bars correspond to the ischemic phases.

The changes in BF show a comparable course for both groups. Significant changes with maxima compared with baseline occurred right at the beginning of the first cycle of ischemia among both groups (T vs. baseline: +70%, *p* = 0.001; T ^e^ vs. baseline: +159%; *p* = 0.001) but the effect decreased at the second (T vs. baseline: +25%, *p* = 0.037; T ^e^ versus baseline: +108%, *p* < 0.001) and third (T vs. baseline: +34%, *p* = 0.026; T ^e^ vs. baseline: +114%, *p* = 0.002) ischemic cycle. Overall, BF at the remote site during ischemia was higher than in reperfusion in both groups at every time point. When concentrating on the reperfusion cycles only, the mean increase in BF for the control was +28% in comparison to the experimental group (+82%). Table 2 gives an illustration of all BF measurements, the course of BF is shown in Fig. 2 . Although the BF in the experimental group at the remote arm is higher compared with control at every time point, it only reached statistical significance in the beginning (T _m2_ : 1.48 ± 1.07 vs. T ^e^_m2_ : 2.20 ± 1.46, *p* = 0.010; T _m4_ : 1.33 ± 0.54 vs. T ^e^_m4_ : 1.93 ± 0.91, *p* = 0.017; T _m5_ : 1.25 ± 0.57 vs. T ^e^_m5_ : 2.08 ± 1.03, *p* = 0.002).

### Oxygen Saturation

A repeated measures ANOVA with a Greenhouse–Geisser correction determined that mean performance levels showed a statistically significant difference between SO _2_ measurements versus baseline for group T ( *F* [1.34, 4.15] = 7.77, *p* < 0.001) and group T ^e^ ( *F* [1.27, 3.54] = 8.62, *p* < 0.001). Baseline measurements were not reached in either of the two groups throughout the measurements.

**Fig. 3:**
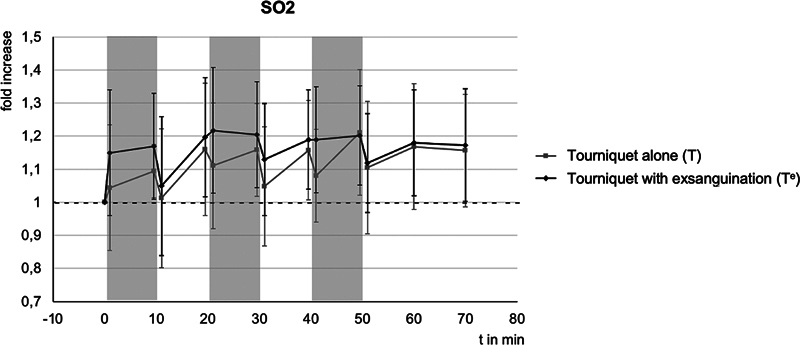
Relative changes of oxygen saturation of hemoglobin (SO _2_ ) compared with baseline, with standard deviation - - -, baseline (1 = 100%), gray bars correspond to the ischemic phases.

The increase in SO _2_ was higher in the experimental group T ^e^ than in the control group except at the end of the last ischemia cycle (T _m10_ : 1.21 ± 0.19 and T ^e^_m10_ : 1.20 ± 0.15, respectively) but diminished over time. The differences between the groups, however, were lower compared with BF and did not reach significance for most of the measurements—only for the beginnings of the three ischemia cycles (T _m1_ : 1.04 ± 0.19 vs. T ^e^_m1_ : 1.15 ± 0.19, *p* = 0.037; T _m5_ : 1.11 ± 0.19 vs. T ^e^_m5_ : 1.22 ± 0.19, *p* = 0.037; T _m9_ : 1.08 ± 0.14 vs. T ^e^_m9_ : 1.19 ± 0.16, *p* = 0.030). After application of the RIC protocol course and difference in SO _2_ levels is out. Table 3 gives an illustration of all measurements. The course of SO _2_ is shown in Fig. 3 .

### Relative Amount of Hemoglobin

**Fig. 4:**
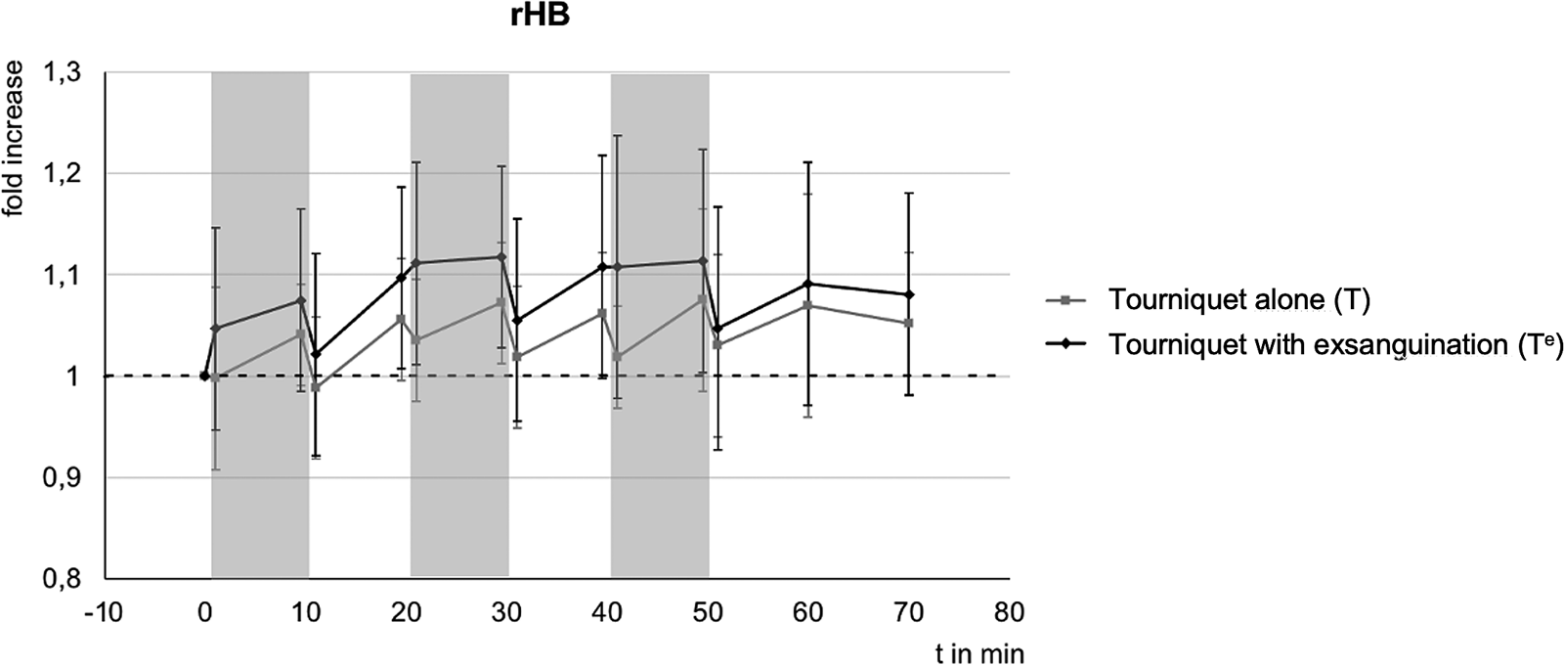
Relative changes of the relative amount of hemoglobin (rHb) compared with baseline, with standard deviation - - -, baseline (1 = 100%), gray bars correspond to the ischemic phases.

The rHb increased in both groups in each ischemic phase, then fell with the beginning of reperfusion, and increased again at the end of the respective reperfusion phase. A repeated measures ANOVA with a Greenhouse–Geisser correction determined that rHb was significantly increased in control group T ( *F* [0.275, 0.95] = 6.95, *p* < 0.001) as well as experimental group T ^e^ ( *F* [0.450, 1.42] = 7.60, *p* < 0.001) compared with baseline measurements. In the first reperfusion phase of the control group, rHb dropped once below the baseline, which was not statistically significant (mean 0.99, SD 0.07, *p* = 0.437). Apart from that, the rHb values remained consistently above the baseline. Values were higher among the experimental than in the control group. However, the MWU test did not show significant differences except for the second and third ischemia cycles (T _m5_ : 1.04 ± 0.06 vs. T ^e^_m5_ : 1.11 ± 0.10, *p* = 0.009; T _m6_ : 1.07 ± 0.06 vs. T ^e^_m6_ : 1.12 ± 0.09, *p* = 0.038; T _m9_ : 1.02 ± 0.05 vs. T ^e^_m9_ : 1.11 ± 0.13, *p* = 0.007). Table 4 gives an illustration of all measurements; the course of rHb is shown in Fig. 4 .

## Discussion

RIC is an effective strategy to protect tissue against ischemia–reperfusion injury. Despite great effort in developing the capabilities of RIC within the last few years, especially in major abdominal or vascular and heart surgery, today's clinical transfer in microsurgery is still pending. 27 28 It has been shown that RIC has a beneficial effect on microcirculation in human subjects. 18 19 20 22 27 29 In the clinical setting of a plastic surgery facility, RIC pre-, peri-, and postinterventional is applicable, for example, Sogorski et al in 2022 found an improvement in microcirculation in perforator flaps in a postconditioning setting of 20 patients. 30 But studies published so far are heterogeneous regarding the applied RIC protocol. For example, they vary between three and four cycles of 5- to 10-minute periods of upper extremity occlusion and reperfusion. 31 Therefore, this study was designed to make a contribution to the development of universal protocols for potential use in everyday clinical practice, considering the intensity of the ischemic stimulus as a contributing factor for the efficiency of RIC. Although the measurements for the experimental group were higher than those of the control group throughout the study, we could not provide statistical significance.

The O2C device is an established, simple, and non-invasive method and has been used in various clinical studies investigating cutaneous microcirculation. 19 20 22 29 30 31 32 33 Our data show that RIC in three cycles of ischemia for 10 minutes is capable of improving cutaneous microcirculation (BF, SO _2_ , and rHb) as previously shown in other studies. 18 19 20 22 Although not statistically significant in comparison of the two tourniquet groups with and without exsanguination, the results are very promising. The changes compared with baseline were most pronounced for the BF. This is in-line with the results of Kolbenschlag et al 19 20 as well as Sogorski et al, 22 where the post-RIC in group 2 (similar to our protocol) was +53.5% compared with our measurements of +31% in the control and even +71% in the intervention group, respectively.

To investigate the influence of different ischemic stimuli on RIC, we compared a protocol using a tourniquet with (T ^e^ , experimental group) and without (T, control group) exsanguination. Used in everyday clinical practice during limb surgery, the additional use of exsanguination by wrapping the limb with an Esmarch bandage before closing the tourniquet reduces the remaining blood by more than half compared with the sole use of a tourniquet. 34 It leads to less remaining oxygen in peripheral tissue and is a strong ischemic stimulus. We hypothesized that a more intense ischemic stimulus could enhance the RIC effects on dermal microcirculation even further. A comparison of the control and experimental group showed more elevated relative changes in microcirculatory values in the experimental group, T ^e^ , compared with those of the control at most of the measurement time points, but did not show statistical significance. The few significant differences between the groups that occurred were almost exclusively during the ischemic phases. This could be explained by a higher blood volume available in the experimental group due to the contralateral arm's exsanguination.

By analyzing the course of the curve of BF, the maximum increase in experimental group T ^e^ is detected during the first cycle of ischemia, which aligns with findings from other studies. 35 RIC effects on the BF are mediated via complex signals to the vascular endothelium with subsequent vasodilation, with nitrite and nitric oxide (NO) playing an important role. 14 15 16 36 RIC likely initiates a sophisticated intra- and extracellular cascade leading to an increased synthesis of endogenous NO via the increased expression of the enzymes endothelial nitric oxide synthase (eNOS) and iNOS (nitric oxide synthase in macrophages and monocytes). 21 Besides a systemic release of humoral factors, 7 8 9 the neural pathway activation with involvement of protein kinase C is also discussed to play a role in the signal cascade. 35 In a publication by Ederer et al, a comparable dynamic of BF was recorded, although the peripheral nerve pathways on the arm used for conditioning were inhibited by plexus block. 32 The pressure of the cuff may address nerve tracts not affected by the plexus block. In addition, the plexus block itself causes peripheral vasodilation by the response of the autonomic nervous system. 37

Tanpowpong et al found that tourniquet tolerance was significantly lower when exsanguination was used compared with limb elevation. 38 On the one hand, this could explain why the experimental group T ^e^ , although not statistically significant, showed a higher increase in microcirculation than the control. On the other hand, it could explain why the BF was more pronounced during the ischemic phases. Jones et al. were able to determine a cardioprotective effect via C-fibers' activation by applying capsaicin in a mouse model. 39 On the contrary, for the cutaneous microcirculation in humans, Rothenberger et al could not show remote effects. 33 However, the stable circulatory parameters detected and the fact that all subjects tolerated the RIC protocol contradict that merely pain is responsible for the effects. But since we did not record pain levels in our study, the possible interference of lower tourniquet tolerance in the experimental group cannot be ruled out as a contributing factor.

The present study showed a significant increase in BF as well as SO _2_ and rHb after the first cycle of ischemia compared with the baseline of both groups. This is in-line with other publications, which also recorded a significant increase in BF immediately after the first cycle. 18 19 22 In the literature, the number and duration of ischemia cycles are controversially discussed in other specialist areas. Usually, three to four cycles of 5 to 10 minutes of ischemia and reperfusion are described as effective. 14 40 However, in isolated cases, cardioprotective effects could be observed after a single ischemia cycle. 41 42 With regard to the cutaneous microcirculation in humans, Sogorski et al showed a dose-dependent effect depending on the number of applied cycles, which lasted up to 4 hours. 22 Since further increase of RIC's effect beyond a certain neurohumoral threshold is not to be expected, they recommend an RIC protocol of three cycles, each consisting of 10 minutes of ischemia/10 minutes of reperfusion, which we used for our study.

The present study is the first to compare the ischemic stimulus's intensity through exsanguination in RIC. Studies published so far examined the influence of the amount of ischemic tissue mass on the effects of RIC. 19 43 Kolbenschlag et al compared ischemic conditioning using a tourniquet of an arm and a leg, respectively, and concluded that an increase in the effector organ's mass had no positive influence on the increase in microcirculation through RIC. 19 Johnsen et al, who compared the cardioprotective effects of conditioning through ischemia of one hindlimb with ischemia of both hindlimbs in an animal model, also found that the reduction in infarct size due to RIC does not depend on the mass of the effector organ. 43 Since we could not find statistically significant differences in dermal BF, it seems probable that neither the intensity of the ischemic stimulus nor the organ mass significantly influences RIC effects.

In our study, we included young and healthy volunteers. Comparable distribution of age, gender, and BMI can be found in the collectives of studies alike regarding the effects of RIC on cutaneous microcirculation. 18 19 21 22 Nevertheless, limitations have to be taken into account in translating the findings of this study to an older and prediseased patient collective. Although a clear trend of superiority in additional exsanguination to the application of a tourniquet is visible, it might also be that our study is statistically underpowered. A larger sample size could have revealed more statistically significant differences. We, therefore, recommend that future studies include larger and more diverse populations to confirm these results and to further explore potential clinical benefits in patient cohorts, including the optimization and standardization of RIC protocols in patient care.

### Conclusion

RIC improves cutaneous microcirculation in healthy subjects. The intensity of the ischemic stimulus using exsanguination in addition to a tourniquet in RIC protocols does not significantly enhance the effect on cutaneous microcirculation, although the relatively small sample size may limit statistical power. Our findings provide an additional aspect to consider in standardizing RIC protocols in future studies. Further clinical studies are necessary to investigate the effects of RIC on cutaneous microcirculation in cases of flap surgery during the pre- and postoperative course of treatment.
